# Closed-loop oxygen control improves oxygen therapy in acute hypoxemic respiratory failure patients under high flow nasal oxygen: a randomized cross-over study (the HILOOP study)

**DOI:** 10.1186/s13054-022-03970-w

**Published:** 2022-04-14

**Authors:** Oriol Roca, Oriol Caritg, Manel Santafé, Francisco J. Ramos, Andrés Pacheco, Marina García-de-Acilu, Ricard Ferrer, Marcus J. Schultz, Jean-Damien Ricard

**Affiliations:** 1 Servei de Medicina Intensiva, Hospital Universitari Vall d’Hebron and Institut de Recerca Vall d’Hebron, Pg Vall d’Hebron 119-129, 08035 Barcelona, Spain; 2 Centro de Investigación Biomédica en Red de Enfermedades Respiratorias (CibeRes), Instituto de salud Carlos III, Madrid, Spain; 3 Department of Intensive Care, Amsterdam University Medical Centers, Location ‘AMC’, Amsterdam, The Netherlands; 4 Nuffield Department of Medicine, University of Oxford, Oxford, UK; 5 Mahidol University, Bangkok, Thailand; 6 Service de Médecine Intensive Réanimation, DMU ESPRIT, Hôpital Louis Mourier, APHP, Université Paris Cité, Colombes, France; 7 INSERM, IAME UMR1137, Paris, France

**Keywords:** Closed-loop oxygen control, Automatic oxygen titration, High-flow nasal oxygen, Nasal high-flow, High flow nasal cannula, Acute respiratory failure

## Abstract

**Background:**

We aimed to assess the efficacy of a closed-loop oxygen control in critically ill patients with moderate to severe acute hypoxemic respiratory failure (AHRF) treated with high flow nasal oxygen (HFNO).

**Methods:**

In this single-centre, single-blinded, randomized crossover study, adult patients with moderate to severe AHRF who were treated with HFNO (flow rate ≥ 40 L/min with FiO_2_ ≥ 0.30) were randomly assigned to start with a 4-h period of closed-loop oxygen control or 4-h period of manual oxygen titration, after which each patient was switched to the alternate therapy. The primary outcome was the percentage of time spent in the individualized optimal SpO_2_ range.

**Results:**

Forty-five patients were included. Patients spent more time in the optimal SpO_2_ range with closed-loop oxygen control compared with manual titrations of oxygen (96.5 [93.5 to 98.9] % vs. 89 [77.4 to 95.9] %; *p* < 0.0001) (difference estimate, 10.4 (95% confidence interval 5.2 to 17.2). Patients spent less time in the suboptimal range during closed-loop oxygen control, both above and below the cut-offs of the optimal SpO_2_ range, and less time above the suboptimal range. Fewer number of manual adjustments per hour were needed with closed-loop oxygen control. The number of events of SpO_2_ < 88% and < 85% were not significantly different between groups.

**Conclusions:**

Closed-loop oxygen control improves oxygen administration in patients with moderate-to-severe AHRF treated with HFNO, increasing the percentage of time in the optimal oxygenation range and decreasing the workload of healthcare personnel. These results are especially relevant in a context of limited oxygen supply and high medical demand, such as the COVID-19 pandemic.

*Trial registration* The HILOOP study was registered at www.clinicaltrials.gov under the identifier NCT04965844.

**Supplementary Information:**

The online version contains supplementary material available at 10.1186/s13054-022-03970-w.

## Background

In patients with acute hypoxemic respiratory failure (AHRF), high flow nasal oxygen (HFNO) has several physiological benefits [1–3] and its use may reduce the need for intubation [4]. Optimal flow settings are unknown and are essentially based on expert recommendations [5]. Current guidelines recommend adjusting the fraction of inspired oxygen (FiO_2_) to maintain oxygenation within the predefined target range, which may vary depending on the risk of hypercapnia and the presence of a medical history of chronic respiratory failure [6].

The deleterious effects of hypoxemia are long recognized. Harmful effects of hyperoxemia are less often considered, but include vasoconstriction, inflammation, and oxidative stress. Several studies have suggested hyperoxemia is associated with poor hospital outcomes in patients under invasive ventilation [7–10], as well as patients receiving non-invasive ventilation [11]. Maintaining oxygenation within a given target range, preventing both hypoxemia and hyperoxemia, requires intensive monitoring and frequent manual adjustments of the FiO_2_ [12], which is time-consuming and therefore often impractical in an era of limited staff resources.

Recent studies found closed-loop oxygen delivery system with automated oxygen titration to be associated with more time spent within the SpO_2_ target range in patient admitted to an emergency department [13], in patients with chronic respiratory disease with exercise-induced desaturation [14], in surgical patients after extubation [15], and in patients weaning from invasive ventilation [16]. Thus far, only one study has been performed in patients with mild AHRF treated with HFNO [17]. It is worth noting that, given the greater FiO_2_ achieved with HFNO in comparison with conventional oxygen therapy, automated oxygen titration could be particularly useful in HFNO patients to prevent hyperoxemia.

Lastly, the COVID-19 pandemic has shown the considerable potential for HFNO to curtail the need for invasive mechanical ventilation [18–20] but has also simultaneously highlighted the overwhelming increase in oxygen demand and the tremendous inequities worldwide in terms of access to oxygen, some countries unable to meet oxygen demands. Limiting both times on HFNO and excessive FiO_2_ could contribute to reduce oxygen consumption.

The objective of this randomised clinical study was to assess the performance of closed-loop oxygen control in critically ill patients with moderate to severe AHRF treated with HFNO. We hypothesized that closed-loop oxygen control increases the time spent within a predefined SpO_2_ range and limits unnecessary exposure to high FiO_2_.

## Methods

### Study design

This is single-centre, single-blinded, randomised crossover trial of closed-loop oxygen control versus manual oxygen titration during HFNO. The study enrolled patients in the Hospital Universitari Vall d’Hebron, Barcelona, Spain, between April 19, 2021, to Aug 18, 2021.

### Patients

Patients were eligible for participation if admitted to ICU and receiving HFNO at a flow rate ≥ 40 L/min with FiO_2_ ≥ 0.30, and expected to receive HFNO for at least 8 h after randomisation. Patients aged < 18 years, pregnant women, patients with an indication or at high risk for immediate intubation, and patients with an indication for non-invasive ventilation were excluded. In addition, we excluded patients that were hemodynamically unstable, patients with severe acidosis, patients with poor SpO_2_ signal, and patients with chronic or acute dyshaemoglobinaemia. We also excluded tracheostomised patients. Patients previously enrolled in this trial or enrolled in another interventional study could not participate.

### Randomisation and masking

Patients were randomised to start with a 4-h period of closed-loop oxygen control or 4-h period of manual oxygen titration, after which each patient was switched to the alternate therapy. Randomization was 1:1, with blocks of 4, using a randomization list that was computer-generated and incorporated into the Research Electronic Data Capture (REDCap) [21] study electronic database. Patients remained unaware of the way oxygen was titrated. Due to the intervention healthcare staff could not be blinded.

### Procedures

After randomisation, the physician in charge decided on the individualized optimal SpO_2_ range for each patient, according to current clinical situation and the medical history. These ranges were 94 to 98%, 92 to 96%, 90 to 94% or 88 to 92%.

With closed-loop oxygen control, there was an automatic adjustment of the FiO_2_ to maintain the patient's SpO_2_ in a predefined target range. The SpO_2_ target range was defined by setting four different cut-offs: an upper and a lower ‘optimal’ cut-off, and an upper and lower ‘suboptimal’ cut-off. These thresholds were different according to the defined target range. The SpO_2_ values within the target thresholds were defined as ‘optimal’. The SpO_2_ values outside the target but within the emergency thresholds were defined as ‘suboptimal’. And those values outside emergency thresholds were considered as out-of-range SpO_2_ values (Additional file 1: Table S1). When the SpO_2_ is in the target range, the controller fine-tunes the FiO_2_ setting to get the patient’s SpO_2_ to the middle of the target range. A detailed description of the device set-up and the way it functions, including FiO_2_ adjustment algorithm and alarm setting are shown in Additional file 1: Table S2. Manual adjustments were allowed in the closed-loop oxygen control group as a patient safety measure.

With manual oxygen titrations, the healthcare personnel was also aware of the optimal SpO_2_ target range. All FiO_2_ adjustments were performed by the physicians and nurses when they observed that the SpO_2_ was not in the predefined target range.

Clinical variables, including systemic blood pressure, heart rate, respiratory rate, SpO_2_, and patient comfort, and HFNO settings were recorded at the start and at the end of each period. FiO_2_, SpO_2_, flow, and temperature were continuously recorded every second using a memory box that was connected to the ventilator via the RS-232 interface port. Device alarms and settings were also recorded with a memory box.

After the first 4 h were completed, a washout period was established for 15 min, after which the patient was switched to the second period with the alternate oxygen titration strategy.

Patient care and standard activities, such as eating or physiotherapy, were uninterruptedly performed as usual, and at random in either period.

### Definitions

Every recorded value of SpO_2_ was classified as either ‘optimal’ when within the individualized SpO_2_ range, ‘suboptimal’ when outside the optimal SpO_2_ range, but inside the suboptimal SpO_2_ range, as shown in Additional file 1: Table S1, or out of range boundaries when outside the suboptimal SpO_2_ range.

### Study endpoints

The primary objective of the study was to assess the efficacy of the closed-loop oxygen control. The primary endpoint measure was the percentage of time spent in the individualized optimal SpO_2_ range. Secondary endpoints included the percentage of time spent in suboptimal ranges, the time spent out of range, the percentage of time with the SpO_2_ signal available, the mean SpO_2_, SpO_2_/FiO_2_, and the ROX index ([SpO_2_/FiO_2_]/respiratory rate) [22, 23], as well as the percentage of time with SpO_2_ below 88% and 85% and number of events of SpO_2_ below 88% and 85%, respectively. The percentage of time with FiO_2_ below 0.4 and above 0.6, the number of manual adjustments required per hour (in manual oxygen titration period), the number and frequency of alarms, and the comfort score assessed by the visual analogic scale from 0 to 10, were also secondary endpoints.

### Sample size estimation

Based on the assumption that closed-loop oxygen control would increase the percentage of time spent with SpO_2_ in optimal range from 51 ± 30 to 81% [13], with power and significance set at 0.80 and 0.05, respectively, 45 patients were required. To account for potential dropouts, defined as a patient who required either non-invasive ventilation or intubation during the course of the study, consent withdrawal by patient or family, poor quality of the SpO_2_ signal for > 1 h during one of the study periods, or technical problem in recording, a sample of 50 patients was initially estimated.

### Statistical analysis

Continuous data were tested for normality using Shapiro–Wilk test. According to their distribution, variables are presented as mean ± standard deviation (SD) or median (IQR, interquartile range) and compared using *t*-test or Wilcoxon test.

Difference estimates are shown as mean of differences in normally distributed variables or as median of differences using the Hodges–Lehman estimator in non-normally distributed variables [24]. Discontinuous data are compared using Chi-square test or Fisher’s exact test, as appropriate.

In order to assess the reproducibility and robustness of closed-loop oxygen control, a sensitivity analysis was performed for the primary endpoint by the severity of the respiratory insufficiency, using the FiO_2_ and the ROX index value at the time for severity classification. We also performed two sensitivity analysis according to the aetiology of AHRF, i.e., AHRF due to coronavirus disease 2019 (COVID-19) versus AHRF due to another cause, and according to the individualized optimal SpO_2_ ranges.

A *p* < 0.05 was considered significant. Statistical analysis was performed using R (version 3.5.2) [25]. We used the built-in packages, ggplot2 [26], tidyverse [27] and epitools [28].

## Results

**Fig. 1:**
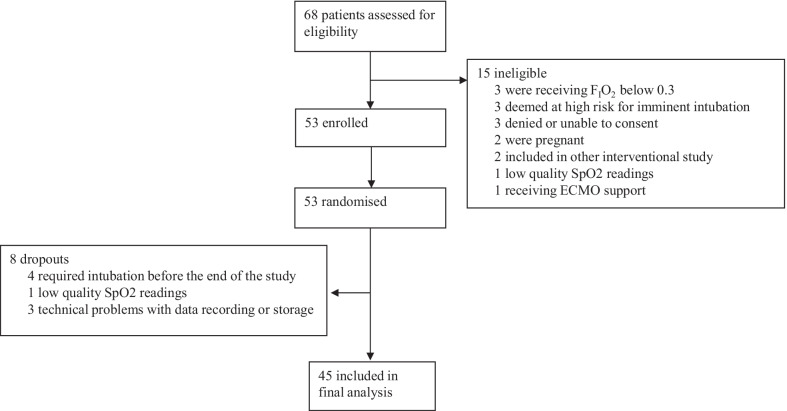
Flow chart of the patients screened, randomized and included

Between April 19, 2021, to Aug 18, 2021, 68 patients were screened for eligibility. Main reason for exclusion was receiving and FiO_2_ < 0.3 and high risk of intubation (Fig. 1). As we had a higher dropout rate than expected, we enrolled and randomised 53 patients of which 45 patients had readable data. Median age was 49 (40 to 62) years, 24 (53.3%) were male. Baseline characteristics and clinical outcomes are presented in Table 1. The majority of patients had AHRF due to COVID-19, and 41 (92.2%) patients received HFNO for de novo AHRF. The median SpO_2_/FiO_2_ at the time of inclusion was 192 (162 to 216). No differences in clinical variables were observed at the start of each study period (Table 2). Average of the total recorded time (hh:mm:ss) was 03:59:45 and 04:03:13 with 99% and 99% of time with valid SpO_2_ signal for closed-loop oxygen control and manual titrations of oxygen, respectively.

**Table 1:** Baseline characteristics of the study cohort

Variables	Median (IQR) or mean (SD) or n (%)
Age	49 (40 to 62)
Sex (male)	24 (53.3%)
Comorbidities	
Hypertension	11 (24.0%)
Diabetes	7 (15.5%)
Immunosupression^a^	10 (22.2%)
Chronic respiratory disease^b^	6 (13.3%)
Reason for admission	
COVID-19 pneumonia	36 (80.0%)
Non-COVID pneumonia	5 (11.1%)
Other	4 (8.8%)
Type of acute respiratory failure	
De novo AHRF	41 (92.2%)
Postextubation support	4 (8.8%)
HFNO characteristics at inclusion	
Hours of HFNO therapy	24 (10 to 48)
F_I_O_2_	0.50 (0.45 to 0.60)
Flow (L/min)	50 (40 to 50)
Severity scores	
SAPS3	45 (39 to 52)
APACHE II	8.5 (5.0 to 15.3)
SOFA	3 (3 to 3)
Outcomes	
ICU length of stay (days)	8 (5 to 15)
Need for mechanical ventilation	12 (26%)
Days of mechanical ventilation	9 (8 to 14)
ICU mortality	2 (4.4%)
Hospital length of stay (days)	16 (9 to 30)
Hospital mortality	4 (4.4%)

IQR: interquartile range; SD: standard deviation; COVID-19: coronavirus-2 disease; AHRF: acute hypoxemic respiratory failure; HFNO: high flow nasal oxygen; SAPS3: Simplified Acute Physiology Score III; APACHE II: Acute Physiology And Chronic Health Evaluation II; SOFA: Sequential Organ Failure Assessment; ICU: Intensive Care Unit

^a^Includes patients with solid or hematologic neoplasms, organ transplant or inflammatory disease receiving immunosuppressing treatment

^b^Includes chronic obstructive pulmonary disease (COPD), asthma, interstitial disease and obstructive sleep apnea syndrome (OSAS)

**Table 2:** Clinical variables at the beginning of each period

Variables	Closed-loop oxygen control	Manual oxygen titration
Heart rate (bpm)	71 (60 to 83)	75 (61 to 83)
Mean blood pressure (mmHg)	86.7 (81.3 to 93.3)	89 (82.7 to 95.3)
Respiratory rate (breaths/min)	19 (18 to 24)	20 (18 to 22)
SpO_2_ (%)	96 (94 to 97)	96 (95 to 97)
FiO_2_	0.50 (0.40 to 0.60)	0.45 (0.40 to 0.60)
ROX	10.26 (3.22)	10.25 (2.87)
Comfort (VAS)	8 (7 to 9)	8 (7 to 9)

Variables are presented as median (IQR) or mean (SD)

IQR: interquartile range; SD: standard deviation. SpO_2_: pulse oximetry; F_I_O_2_: fraction of inspired oxygen; ROX: respiratory rate and oxygenation index ([SpO_2_/F_I_O_2_]/RR); VAS: visual analogue scale

**Fig. 2:**
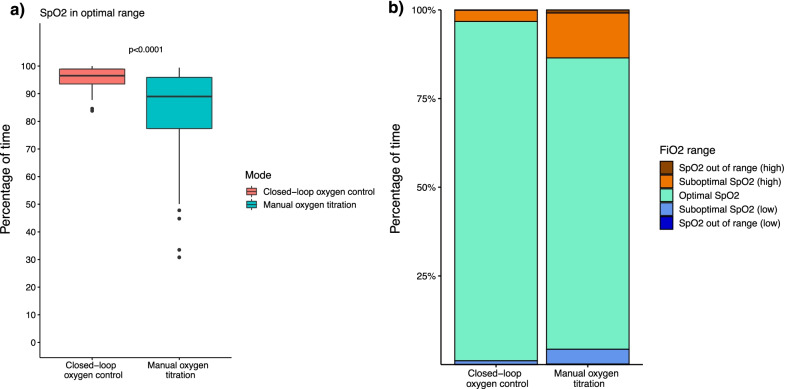
Comparison of SpO_2_ values between closed-loop oxygen control and manual oxygen titration. **A** Percentage of time in the optimal range. **B** Percentage of time spent in optimal, suboptimal ranges and out of range. For illustrative purposes, the height of each bar of **B** represents the mean of the values

With closed-loop oxygen control, patients spent more time in the optimal SpO_2_ range compared with manual titrations of oxygen (96.5 [93.5 to 98.9] % vs. 89 [77.4 to 95.9] %; *p* < 0.0001) (difference estimate, 10.4 (95% confidence interval 5.2 to 17.2) (Fig. 2). With closed-loop oxygen control, patients spent less time in the suboptimal range, both above (0.6 [0.1 to 1.1] % vs. 1.0 [0.1 to 4.0] %; *p* = 0.0053) and below (2.3 [0.8 to 5.2] % vs. 4.2 [1.1 to 16.4] %; *p* = 0.0004) the cut-offs of the optimal SpO_2_ range (Table 3). With closed-loop oxygen control, patients spent less time beyond the suboptimal range. The number of events of SpO_2_ < 88% and < 85% were not significantly different between groups.

**Table 3:** Primary and secondary outcomes

Variable	Closed-loop oxygen control	Manual oxygen titration	Difference estimate (95% CI) or mean difference or OR	*p* value
*Primary outcome*				
Time in optimal SpO_2_ range (% of the total recording time)	96.5 (93.5 to 98.9)	89.0 (77.4 to 95.9)	10.40 (5.20 to 17.20)	< 0.0001
*Secondary outcomes*				
Time in suboptimal SpO_2_ range (% of the total recording time)				
Low	0.6 (0.1 to 1.1)	1.0 (0.1 to 4.0)	− 1.20 (− 4.95 to − 0.25)	0.0053
High	2.3 (0.8 to 5.2)	4.2 (1.1 to 16.4)	− 5.25 (− 14.45 to − 1.40)	0.0004
Time out of SpO_2_ range (% of the total recording time)				
Low	0 (0 to 0)	0 (0 to 0.1)	− 0.10 (− 0.35 to 0.10)	0.3972
High	0 (0 to 0.1)	0 (0 to 0.3)	− 0.50 (− 2.15 to − 0.05)	0.0166
Percentage of time with SpO_2_ signal available	99.7	99.8	− 0.10 (− 0.35 to 0.30)	0.6235
Mean SpO_2_/F_I_O_2_ during the recording time	206 (176 to 254)	210 (165 to 235)	8.21 (− 7.08 to 23.38)	0.2715
ROX index				
At the beginning	10.30 (3.22)	10.30 (2.87)	Mean difference 0.009 (− 0.83 to 0.85)	0.9829
At the end	11.50 (4.48)	10.60 (3.52)	Mean difference 0.88 (− 0.46 to 2.23)	0.1916
ΔROX^a^	1.20 (4.20)	0.33 (2.37)	Mean difference 0.87 (− 0.77 to 2.52)	0.2917
Time with SpO_2_ < 88% (% of the total recording time)	0 (0 to 0)	0 (0 to 0)	− 0.10 (− 0.20 to 0.05)	0.2470
Time with SpO_2_ < 85% (% of the total recording time)	0 (0 to 0)	0 (0 to 0)	− 0.10 (− 0.55 to 0.10)	0.3741
Number of events (SpO_2_ < 88%) per hour	0 (0 to 0)	0 (0 to 0)	0.05 (− 0.38 to 0.38)	0.9055
Number of events (SpO_2_ < 85%) per hour	0 (0 to 0)	0 (0 to 0)	− 0.25 (− 0.51 to 0.25)	0.5862
Mean F_I_O_2_ during the recording time	0.5 (38.1 to 55.8)	0.5 (40.0 to 56.6)	0.75 (− 2.80 to 4.55)	0.6155
Time in different F_I_O_2_ (% of the total recording time)				
F_I_O_2_ < 0.4	21.7 (0 to 66.0)	0 (0 to 0)	32.29 (12.05 to 50.30)	0.0025
F_I_O_2_ 0.4–0.6	46.0 (10.3 to 70.2)	100 (63.2 to 100)	− 41.08 (− 54.80 to − 23.80)	< 0.0001
F_I_O_2_ > 0.6	4.2 (0.0 to 29.8)	0.0 (0.0 to 0.0)	16.43 (6.40 to 35.20)	< 0.0001
Number of manual adjustments per hour	0 (0 to 0)	0.21 (0 to 0.47)	− 0.49 (− 0.72 to − 0.36)	< 0.0001
Patients with ≥ 1 alarms/h for low SpO_2_	16 (35.6%)	22 (48.9%)	OR 0.57 (0.25–1.34)	0.2003
Patients with ≥ 1 alarms/h for high SpO_2_	1 (2.2%)	1 (2.2%)	OR 1.00 (0.06–16.49)	1.0000
ΔComfort score^a^	0 (0 to 1.0)	0 (− 0.25 to 0.125)	1.0 (0 to 1.50)	0.0468

Results are shown as median (interquartile range, IQR) or as mean (standard deviation, SD). Wilcoxon or student’s t test were performed depending on each variable distribution according to the Shapiro–Wilk test. For alarms, a Fisher’s exact test was performed

*OR* odds ratio, *95% CI* 95% confidence interval

^a^ΔROX and ΔComfort score were calculated as the value at the end of the period minus the value at the beginning of the period. SpO_2_: pulse oximetry; F_I_O_2_: fraction of inspired oxygen; ROX: respiratory rate and oxygenation index ([SpO_2_/F_I_O_2_]/RR)

Mean FiO_2_ used during each period and SpO_2_/FiO_2_ ratio was not different between closed-loop oxygen control and manual oxygen titration. Time spent at different FiO_2_ levels, however, was different (Table 3, Additional file 1: Fig. S1). With closed-loop oxygen control, FiO_2_ varied much more than with manual oxygen titration. The number of manual adjustments was very different between the two approaches. Only one patient during the closed-loop oxygen control required one single manual adjustment of FiO_2_ due to SpO_2_ sensor dislodgement that was solved with SpO_2_ sensor repositioning. The number of alarms for a low or high SpO_2_ was not different between the two approaches.

Oxygen consumption during the time that the patients spent above the optimal SpO_2_ targets was significantly lower with closed-loop oxygen control. (Additional file 1: Table S3).

The improvement in time within optimal SpO_2_ range in closed-loop oxygen control versus manual oxygen titrations remained significant when patients were analysed according to initial FiO_2_ (Additional file 1: Table S4). Interestingly, the effect was stronger in more hypoxemic patients (Additional file 1: Fig. S2). The findings were neither affected by the cause of AHRF nor by the individualised SpO_2_ ranges (Additional file 1: Table S4).

## Discussion

The findings of this study in patients with moderate AHRF treated with HFNC can be summarized as follows: (1) with the use of closed-loop oxygen control, patients spent more time in the optimal SpO_2_ range; (2) the closed-loop oxygen control decreased the time in the suboptimal range both above and below and out-of-range above the target; (3) contrary to what happened with standard of care, closed-loop oxygen control was equally effective regardless the severity of oxygenation impairment, (4) the closed loop oxygen control significantly decreased the number of manual adjustments, reducing the healthcare personnel workload, and (5) significantly decreased oxygen consumption. Given the increasing use of HFNO as highlighted worldwide during the pandemic, the growing concern of hyperoxia in critically ill patients, and the incapability of some countries to meet oxygen demands, our results bear important consequences on routine practice.

The time spent in optimal SpO_2_ target both during closed-loop and during the manual oxygen titration period was considerably higher than previously reported. L’Her et al. [13] reported that patients spent 81% and 51% of time in optimal SpO_2_ range while Harper et al. [17] had 96.2% and 71% in closed-loop and manual oxygen titration periods, respectively. Differences in the study setting and patient’s characteristics may explain these different results. One study was performed on patients admitted to the emergency department who were receiving low flow oxygen [13] while the other study focused on patients with mild AHRF treated with HFNO but who were admitted to medical or surgical wards [17]. In the present study, patients were admitted to an ICU or intermediate care unit with lower nurse-to-patient ratios, increasing the time that they spent within the optimal range in the manual oxygen titration period compared to those previously reported. Interestingly, they presented a higher respiratory impairment with higher needs for FiO_2_ and lower SpO_2_/FiO_2_ ratios at inclusion and during the study period, increasing the risk of being out of the optimal range. Therefore, our results demonstrate that closed-loop oxygen control improves oxygen administration also in patients with moderate to severe AHRF who required higher oxygen requirements than previously studied.

The closed-loop oxygen control decreased the time spent in suboptimal ranges both above and below limits and also the time out of range above the out-of-range limits. Overall, the closed-loop oxygen control had a higher impact in reducing hyperoxia rather than hypoxia, in line with previous results reported in patients treated with low-flow oxygen who were admitted to the emergency department [13], during the early post-extubation period [16], and in COPD patients [29]. The effect of reducing hyperoxia is extremely important for two different reasons. First, hyperoxia has been associated with worse outcomes in patients with acute respiratory distress syndrome [9] and, in the context of the shortage of oxygen that many countries have experienced during the pandemic, it may decrease the oxygen consumption in a patient and at a hospital level. In contrast, no differences were seen in time spent out of range below the limits which makes sense as all patients were admitted in the ICU or intermediate care units and they were closely monitored to prevent any clinically significant desaturation.

To our knowledge, only 2 articles have reported closed-loop oxygen control systems during HFNO therapy. The first study assessed FiO_2_ adjustment during a 6-min walking test (6MWT)-induced desaturation in patients with chronic respiratory diseases [14]. The second study is a recent randomized trial that included 20 patients with mild AHRF [17]. While this study demonstrated that the use of closed-loop oxygen was associated with more time in optimal SpO_2_ range, the median difference was rather small (− 1% for hyperoxia and − 2.4% for hypoxia). Applicability of these results to the ICU setting is limited since, contrary to our study, patients receiving FiO_2_ higher than 0.4 were excluded. Interestingly, our results suggest that the benefit of closed-loop oxygen control could be even more clinically relevant in more hypoxemic patients.

It should be also noted that a median of 0.21 manual adjustments per hour were needed during manual oxygen titration. This would translate into approximately 6 manual changes a day, per patient. COVID-19 pandemic has shown the critical issue of staffing and staff shortage in many hospitals and a tremendous increase in their workload. Importantly, nurse workload is a major challenge in the quality of care and it may have a direct impact on patient outcomes [30] and pre-pandemic studies [31] have shown that objective assessment of nurse workload identified excessive workload compared to the 1:1.5 nurse/patient ratio recommendation. Hence the potential benefits of automated systems that contribute to reduce nurse workload may help improve patients’ outcomes. Of note, the study was performed on ICU patients. Thus, the observed results could be even more important in areas of care with a less favourable nurse-to-patient ratios. The closed-loop oxygen control significantly reduced the number of adjustments needed and therefore, reduced the workload for medical and nursing staff, as shown previously for other automatic oxygen devices in mechanically ventilated patients [12]. These findings are especially relevant in a context of high medical demand, such as the COVID-19 pandemic.

Oxygen consumption has been an important issue during the COVID19 pandemic [32]. Some strategies aiming to reduce the oxygen consumption have been used, such as using more conservative or lower oxygen targets, the use of oxygen-saving systems or oxygen concentrators, such as the Double-Trunk Mask, which, placed over a low-flow oxygen mask, may decrease the total oxygen flow required [33]. We herein report a higher oxygen consumption during manual oxygen titration as compared to automatic closed-loop oxygen control when patients were above the optimal SpO_2_ range. This suggests they consumed an excess of oxygen which is wasted while keeping patients above the optimal SpO_2_ target. Our findings suggest that a closed-loop oxygen system may result in a more economic use of oxygen, with more oxygen spent to avoid desaturation but with less oxygen wasted, keeping the patients longer within the optimal range and decreasing the time patients spent above the optimal SpO_2_ targets. The fact that no significant difference in total oxygen consumption was found might be explained by our relatively short recording time and the fact that the study was underpowered to detect differences in this particular outcome. Thus, it seems reasonable to think closed-loop oxygen control system might be a way to spare oxygen, especially in high oxygen-demand periods such as COVID-19 [32].

Even though there were no differences between periods in the mean F_i_O_2_ during the 4 h of the study, during closed-loop oxygen control, patients spent more time with an F_I_O_2_ below 0.4 and above 0.6 compared with the standard of care period. This result suggests that closed-loop oxygen titration tailors the use of oxygen to patient’s individual needs during the course of the disease better than the manual titrations of oxygen and, therefore a more appropriate and adequate oxygen administration is achieved during closed-loop oxygen control in patients with moderate to severe AHRF.

The present study has important strengths. It was a randomized and cross-over study with each patient serving as its own control and replacing the dropout to finally obtain the estimated sample size. We also used individualized ranges and cut-offs according to the clinical condition of each patient and sensitivity analyses showed that closed-loop oxygen was equally effective regardless of the predefined optimal SpO_2_ target. Additional sensitivity analyses showed that closed-loop oxygen control was equally effective regardless of the aetiology of respiratory failure or the FiO_2_ at the time of enrolment.

In contrast, it has several limitations. It is a single-centre study with impossibility of blinding. Moreover, only a limited time span was analysed (4 h per period). Together with the crossover design, this does not allow for analysis of more clinically significant outcomes, such as days of HFNO therapy or ICU length of stay. However, it is the first time that a closed-loop oxygen control showed to be efficient and safe for patients with moderate to severe AHRF treated with HFNO.

## Conclusions

The present study shows that a closed-loop oxygen control system improves oxygen administration in critically ill patients with moderate-to-severe AHRF treated with HFNO by mainly decreasing the time the patient spent above the limits of the clinically set oxygenation targets. The closed-loop oxygen control was also associated with a lower need for manual oxygen adjustments. These results may have important implications both at the patient level, as it decreases the risk of deleterious effects of hypoxia and hyperoxia, and at the healthcare system level as it decreases the healthcare personnel workload and it might be potentially associated with a less use of oxygen, making it a useful asset for high oxygen-demand periods such as a pandemic.

## Supplementary Information

**Additional file 1.** Supplementary Material.

## Data Availability

The datasets generated during the current study are not publicly available due to patient confidentiality reasons, but are available from the corresponding author upon reasonable request. All requests will also need to be reviewed by our Ethics Committee before any data is shared.

## Abbreviations

AHRF: Acute hypoxemic respiratory failure
HFNO: High flow nasal oxygen
F_I_O_2_: Fraction of inspired oxygen
SpO_2_: Pulse oxymetry
COVID-19: Coronavirus-2 disease
ICU: Intensive care unit
ROX: Respiratory rate and oxygenation index
SD: Standard deviation
IQR: Interquartile range
6MWT: 6-Minute walking test
SAPS3: Simplified Acute Physiology Score III
APACHE II: Acute Physiology and Chronic Health Evaluation II
SOFA: Sequential Organ Failure Assessment
