# Supplementary material for: Closed-loop oxygen control improves oxygen therapy in acute hypoxemic respiratory failure patients under high flow nasal oxygen: a randomized cross-over study (the HILOOP study)
Source: Crit Care. 2022 Apr 14;26:108. doi: 10.1186/s13054-022-03970-w (PMC9008383; doi:10.1186/s13054-022-03970-w)
Supplement: Supplementary file 1 — Additional file 1. Supplementary Material. [file 13054_2022_3970_MOESM1_ESM.docx]

**Supplementary Material:**

**Closed-loop Oxygen Control Improves Oxygen Therapy in Acute Hypoxemic Respiratory Failure Patients under High Flow Nasal Oxygen Therapy––a randomized cross-over study (the HILOOP study)**

Oriol Roca MD, PhD^1,2^, Oriol Caritg MD^1^, Manel Santafè MD^1^,

Francisco J. Ramos MD^1^,Andrés Pacheco MD^1^, Marina García-de-Acilu MD^1^,

Ricard Ferrer MD PhD^1,2^, Marcus J Schultz MD, PhD^3,4,5^, Jean-Damien Ricard MD, PhD^6,7^

[Methods 3](#_Toc91828331)

[Description of the automatic closed-loop device and equipment set-up 3](#_Toc91828332)

[Role of the funding source 3](#_Toc91828333)

[Supplementary tables 4](#_Toc91828334)

[Table S1. Optimal and suboptimal oxygen ranges*.* 4](#_Toc91828335)

[Table S2. Functioning of oxygenation controller software. 5](#_Toc91828336)

[Table S3. Oxygen consumption 6](#_Toc91828337)

[Table S4. Sensitivity analyses. 7](#_Toc91828338)

[Supplementary figures 8](#_Toc91828339)

[Figure S1. Fraction of inspired oxygen use in standard care and closed-loop oxygen control mode. 8](#_Toc91828340)

[Figure S2. Relation between percentage in optimal SpO2 range and patient’s hypoxaemia. 9](#_Toc91828341)

# Methods

## Description of the automatic closed-loop device and equipment set-up

The closed loop oxygen controller is a software option implemented on the commercially available HAMILTON-C1 and HAMILTON-T1 ventilators (Hamilton Medical, Bonaduz, Switzerland) with the latest software version 3.0.0 CE certified. The ventilators have an option board that allows the connection to a pulse oximeter (Masimo Corporation, Irvine, USA). The activation of the Automated F_I_O_2_ option requires the continuous use of the pulse oximeter sensor that has to be connected to the patient’s finger or ear. The Automatic F_I_O_2_ option provides automated adjustment of the ventilator Oxygen setting to maintain the patient's SpO_2_ in a defined target range. When using the software option, the user defines the SpO_2_ target range, as well as the SpO_2_ emergency limits, and the device adjusts the Oxygen setting to keep the patient's SpO_2_ in the target range (Table 1).

The oxygenation controller adjusts the oxygen settings as presented in Table S2. When the SpO2 is below the target range the FiO2 every 30 seconds whereas when the SpO2 is above the target range, the FiO2 increases every minute.

The oxygenation controller is paused when: 1) SpO_2_ monitoring is unavailable, 2) SpO_2_ quality index is low (1 red bar or less), 3) SpO_2_ < 50%, 4) there is an oxygen supply failure, 5) O_2_ enrichment is active, 6) O_2_ sensor calibration is in progress, 7) flow sensor calibration is in progress, 8) leak test is in progress, or 9) the device is in Standby, Safety ventilation, Safety mode, or in the Ambient state. Once the event is resolved or the action is completed, the device resumes automatic adjustment of the oxygen control. Automatic adjustment of the oxygenation controller is disabled when SpO_2_ or F_I_O_2_ monitoring is disabled.

## Role of the funding source

For each patient, an anonymised file containing the recorded data from the high-flow device was sent to Hamilton Medical AG, where this data was transformed into a database of raw data including the main variables and sent back to the investigators for further analysis. Hamilton Medical did not have a role in statistical analysis beyond this point nor data interpretation.

# Supplementary tables

## Table S1. Optimal and suboptimal oxygen ranges*.*

| **Clinical SpO_2_ targets** | **Out of range (low)** | **Suboptimal low** | **Optimal** | **Suboptimal high** | **Out of range (high)** |
| --- | --- | --- | --- | --- | --- |
| 94 – 98 % | < 90% | ≥ 90 - < 94% | 94 – 98% | > 98 - ≤ 99% | 100% |
| 92 – 96% | < 89% | ≥ 89 - < 92% | 92 – 96% | > 96% - ≤ 98% | > 98% |
| 90 – 94% | < 86% | ≥ 86 - < 90% | 90 – 94% | > 94% - ≤ 97% | > 97% |
| 88 – 92% | < 85% | ≥ 85 - < 88% | 88 – 92% | > 92% - ≤ 95% | > 95% |

## Table S2. Functioning of oxygenation controller software.

| **Condition** | **Oxygenation controller action** |
| --- | --- |
| SpO_2_ is in range (between Target-Hi and Target-Lo) | The controller fine-tunes the Oxygen setting to get the patient's SpO_2_ to the middle of the target range. |
| SpO_2_ is low (below Target-Lo and above Emer-Lo) | The controller increases the Oxygen setting every 30s. |
| SpO_2_ is too low (below Emerg-Lo) | The controller increases the Oxygen setting with larger step every 30s. |
| SpO_2_ is high (above Target-Hi and below Emerg-Hi) | The controller decreases the Oxygen setting every minute. |
| SpO_2_ is too high (above Emerg-Hi) | The controller decreases the Oxygen setting with larger step every minute. |
| SpO_2_ measurement is unavailable | The Oxygen control is frozen and is displayed as a red circle together with an alarm |

## Table S3. Oxygen consumption

|  | **Closed-loop oxygen control** | **Manual oxygen titration** | **Difference (95% CI)** | **p-value** |
| --- | --- | --- | --- | --- |
| Total O_2_ consumption (L/h) | 676 (467 to 1091) | 720 (530 to 1062) | 17.24 (-85.14 to 136.85) | 0.6547 |
| O_2_ consumption during the time above the optimal range (L/h) | 18·5 (5.3 to 53.4) | 32·2 (7.3 to 97.5) | -31.61 (-99.85 to -4.50) | 0.0052 |

Oxygen consumption in closed-loop oxygen control versus standard care. Oxygen consumption in hyperoxia corresponds to the litres of oxygen spent during the time above the optimal range (suboptimal high + out of range high). The amount of litres of oxygen used during the study was divided per total recorded time (in hours).

## Table S4. Sensitivity analyses.

|  | **Automatic** | **Standard** | **Difference (95% CI)** | **p-value** | **Automatic** | **Standard** | **Difference (95% CI)** | **p-value** |
| --- | --- | --- | --- | --- | --- | --- | --- | --- |
|  | **F_I_O_2_ ≤ 0.5** (n=27) | | | | **F_I_O_2_ > 0.5** (n=18) | | | |
| Percentage of time within the optimal SpO_2_ range | 97.6  (95.6 to 99.2) | 93.8  (82.3 to 96.2) | 7.61  (2.95 to 15.55) | 0.0001 | 95.2  (91.7 to 98.1) | 81.6  (66.2 to 93.2) | 14.95  (5.65 to 30.20) | <0.0001 |
|  | **COVID-19** (n= 36) | | | | **Other** (n=9) | | | |
| Percentage of time within the optimal SpO_2_ range | 96.4  (93.6 to 99.0) | 88.5  (76.3 to 95.4) | 10.55  (4.2 to 17.35) | <0.0001 | 96.9  (93.2 to 98.8) | 90.3  (82.5 to 96.7) | 8.65  (-0.1 to 28.0) | 0.0547 |
|  | **SpO_2_ target 92 – 96%** (n=28) | | | | **SpO_2_ target 94 – 98%** (n=17) | | | |
| Percentage of time within the optimal SpO_2_ range | 95.5  (92.3 – 97.7) | 81.9  (57.3 – 95.4) | 16.10  (7.90 to 25.70) | 0.0006 | 98.9  (96.9 – 99.7) | 92.2  (87.9 to 95.9) | 5.25  (2.55 to 10.10) | 0.0011 |

Analysis of the primary outcome was performed stratifying the patients by initial F_I_O_2_, COVID-19 status and clinical S_p_O_2_ target range. Results are shown as median (interquartile range, IQR), 95%CI 95% confidence interval. SpO_2_: pulse oximetry; F_I_O_2_: fraction of inspired oxygen.

# Supplementary figures

## Figure S1. Fraction of inspired oxygen use in standard care and closed-loop oxygen control mode.


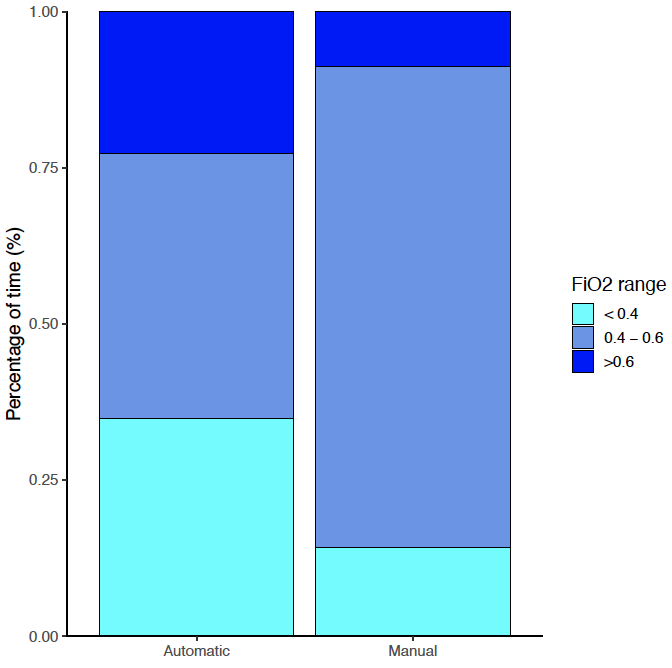


Percentage of time spent with FIO2 < 0.4, between 0.4 and 0.6, and above 0.6. For illustrative purposes, the

height of each bar represents the mean of the values, instead of the median, even though the variable was

analysed as non-parametric in the statistical analysis. FIO2, inspired fraction of oxygen.

## Figure S2. Relation between percentage in optimal SpO2 range and patient’s hypoxaemia.


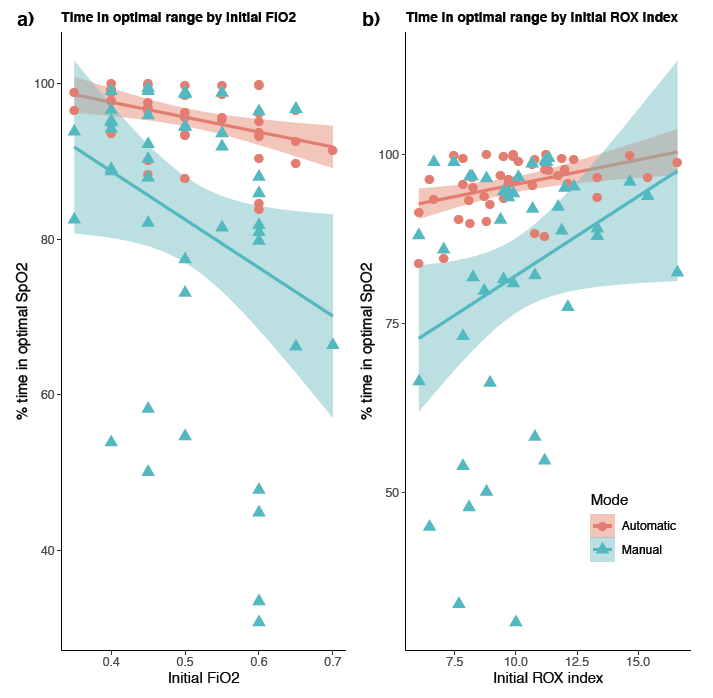


A) According to initial FIO2. B) According to initial ROX index (note that a lower ROX index indicates

more hypoxemia). Regression line with a 95% confidence interval for linear regression.
